# Phase synchronization and measure of criticality in a network of neural mass models

**DOI:** 10.1038/s41598-022-05285-w

**Published:** 2022-01-25

**Authors:** Sheida Kazemi, Yousef Jamali

**Affiliations:** Biomathematics Laboratory, Department of Applied Mathematics, School of Mathematical Sciences, Tarbiat Modares University, Tehran, Iran

**Keywords:** Dynamical systems, Nonlinear phenomena, Phase transitions and critical phenomena

## Abstract

Synchronization has an important role in neural networks dynamics that is mostly accompanied by cognitive activities such as memory, learning, and perception. These activities arise from collective neural behaviors and are not totally understood yet. This paper aims to investigate a cortical model from this perspective. Historically, epilepsy has been regarded as a functional brain disorder associated with excessive synchronization of large neural populations. Epilepsy is believed to arise as a result of complex interactions between neural networks characterized by dynamic synchronization. In this paper, we investigated a network of neural populations in a way the dynamics of each node corresponded to the Jansen–Rit neural mass model. First, we study a one-column Jansen–Rit neural mass model for four different input levels. Then, we considered a Watts–Strogatz network of Jansen–Rit oscillators. We observed an epileptic activity in the weak input level. The network is considered to change various parameters. The detailed results including the mean time series, phase spaces, and power spectrum revealed a wide range of different behaviors such as epilepsy, healthy, and a transition between synchrony and asynchrony states. In some points of coupling coefficients, there is an abrupt change in the order parameters. Since the critical state is a dynamic candidate for healthy brains, we considered some measures of criticality and investigated them at these points. According to our study, some markers of criticality can occur at these points, while others may not. This occurrence is a result of the nature of the specific order parameter selected to observe these markers. In fact, The definition of a proper order parameter is key and must be defined properly. Our view is that the critical points exhibit clear characteristics and invariance of scale, instead of some types of markers. As a result, these phase transition points are not critical as they show no evidence of scaling invariance.

## Introduction

Synchronization is the coordination of one dynamical property by coupling or external force between elements of a system. The simplest of synchronization is full synchronization proposed by Fujisaka and Yamada^1^. In 1996, Rosenblum was the first one who introduced the concept of phase synchronization^2^. Due to this definition, the phase difference is fixed (smaller than 2$\pi$), but amplitudes are not necessarily the same. At least two elements are involved in the synchronization process. To investigate network synchrony, we first assume that *N* oscillators interact with each other. Each oscillator state shows a fixed-point attractor (steady-state), a limit cycle (a closed cycle that shows periodic oscillations), or chaos (a complex orbit that determines aperiodic oscillations). It was demonstrated that networks composed of chaotic neural oscillators tend to display multistability more readily than networks composed of nonchaotic neurons^3^. Kuramoto’s model is a classic example of how collective synchronization emerges spontaneously^4,5^. There have been extensive studies on the coupled Kuramoto model over the last several years, and a detailed review on this issue can be found in^6,7^. In general, networks of oscillators typically exhibit trivial dynamics, known as full synchronization, when no delays are present^5^.

A method for estimating oscillatory activity is the measurement of synchronization in neural signals^8–10^. Studies have examined the physiology of many different types of oscillations in the brain, including theta, gamma, and sleep waves^11^. Different linear (e.g., cross-correlation) or nonlinear (e.g., mutual information) methods to measure rhythmic neural interactions can be used to analyze signals in time and frequency space^12^.

Synchronization measures have been wildly interested in investigating neural dynamics^13–16^. Especially, synchronization phenomena have a substantial role in determining normal^17,18^ and abnormal^19^ brain function and also in a deeper understanding of information processing^20–22^. Moreover, synchronization influences on prediction and detection of some disorders such as epilepsy^23^, Alzheimer^24^, autism^25^, and schizophrenia^26^.

Epilepsy is a common neurological disease worldwide. About 1% of people suffer from epilepsy^27^. Many patients can control their disease by taking medicine, but approximately 30% of them have drug-resistant epilepsy. The dynamics of epilepsy disorder is very complex, and it arises from high synchrony of neuron activities^28^. The significant changes in the brain dynamics during epilepsy can be fatal, and in certain conditions, lead to loss of consciousness, body tremors, and even death. So, enhancing knowledge of the epilepsy mechanism is imperative.

The brain biophysical modeling is an active research area for neuroscientists. In this approach, mathematical and physical tools are used to predict the influence of different biological factors on a complex system^11,29,30^. This attitude causes a profitable framework in neural dynamics modeling with large-scale approaches^31–34^. Brain function can be studied at different scales, from a small patch of the cortex to the largest system. Some cognitive activities such as memory, learning, and perception arise from a mass activity of neurons. So, the population of neurons is coupled together to form a neural mass, and they can model different phenomena and generate a variety of dynamics behaviors. Coupling a collection of the neural mass model into a mesoscale circuit can provide a link between these scales. Moreover, a complementary study^35^ notes that local dynamics play a substantial role in the shaping of large-scale functional brain states^35^.

Neural mass models have a long history. Lopez da silva^36,37^, Jansen and Rit^38,39^, Wendling^40,41^, Wilson–Cowan^42–45^, Freeman^46–48^, and Wong–Wang^49,50^ are some models that investigated the collective behavior of neurons. These models allow researchers to study different rhythms and the transition between normal activity in the brain and epilepsy. Moreover, the complexity of electroencephalogram (EEG) shows the complexity of cortical columns that can transform into a simple circle using neural models, and accordingly, their analysis is conceivable. The comparison of neural mass models has received a lot of attention. Some models have the ability to support partial synchronization, while other models can support scale-free synchronization^51^. Also, in^52^, two neural mass models have been comprised by different measures. Since synchronization is an appropriate measurement for neural dynamics, it has appeared as an ordered parameter in many research^51–57^. In brain networks, the coupling strength between units^54,55^ and the amount of external input of nodes^49,58,59^ are two interesting parameters to be analyzed. Indeed, neural mass models can show different behaviors due to these parameters, and initial conditions vary in each run. Both of them have a significant role in developing a wide range of activities.

Since 1988, the Jansen–Rit model has been discussed analytically and numerically^38,39,60–62^. The purpose of this paper is to analyze the dynamics of the Jansen–Rit model that arose to extend simultaneous electrical activities simulation, especially alpha rhythm, in neural masses. Also, Jansen and Rit demonstrated that their model simulates evoked potential. The disability of making different rhythms, especially onset activity of epilepsy, is the main disadvantage of this model. Various activities such as alpha-like activity and seizure-like activity can be induced by using different inputs to a single Jansen–Rit neural mass model^39^ and two coupled Jansen–Rit neural mass models^60^. Moreover, the concept of criticality was studied in a single column with Jansen–Rit dynamics^63^.

Based on the critical brain hypothesis, the healthy brain function is at or near the transition between different dynamical regimes. It is reported that these critical states have beneficial properties including optimal information transformation and high processing capability^64–67^. There have been many discussions of theoretical aspects of criticality relying on the tools of physics such as correlation functions and critical exponents^68,69^. A complementary perspective to physical theory is dynamical system theory, which studies critical phenomena from a systemic viewpoint. In dynamical systems theory, transitions between regimes in dynamics are modeled mathematically. As a result, phase transitions manifest as so-called bifurcations of system dynamical variables.

To investigate a network of coupled phase oscillators, many research such as^35,58,70^, used empirically measured individual structural connectivity. Structural links of cortical neurons in a network show a small-world topology^71^, so several studies surveyed small-world networks of interconnected neural mass models^44,54,72,73^.

In this paper, we model the brain as a system of coupled oscillators in the mesoscale approach. First, we explain the one-column Jansen–Rit model, and then in a network and alter the external input in four levels. We used four measures to quantify synchronization: Pearson Cross-Correlation, Phase Locking Value, Kuramoto Order Parameter, and Phase Locking Index^12,74^. We discovered that they all produce results with nearly the same quality. So, we chose just one of them (Pearson Cross-Correlation). This network shows a phase transition between physiological and seizure-like behavior in the weak external input level. We considered the coupling coefficient between nodes and synchronization as control and order parameters, respectively. Next, the synchronization diagram according to the coupling coefficient with synchrony measures has been drawn. Finally, to recognize critical behavior, some markers of its such as high fluctuations of synchronization (weak stability) and being on the edge of synchronization, and LRTC (long-range temporal correlation) in the amplitude of neural oscillations, has been selected. Our results show that these phase transition points have some markers of criticality that are a result of the nature of the specific order parameter selected to observe these markers. Our view is that the critical points exhibit clear characteristics and invariance of scale, instead of some types of markers. As a result, these phase transition points are not critical as they show no evidence of scaling invariance.

### Jansen–Rit model

**Figure 1:**
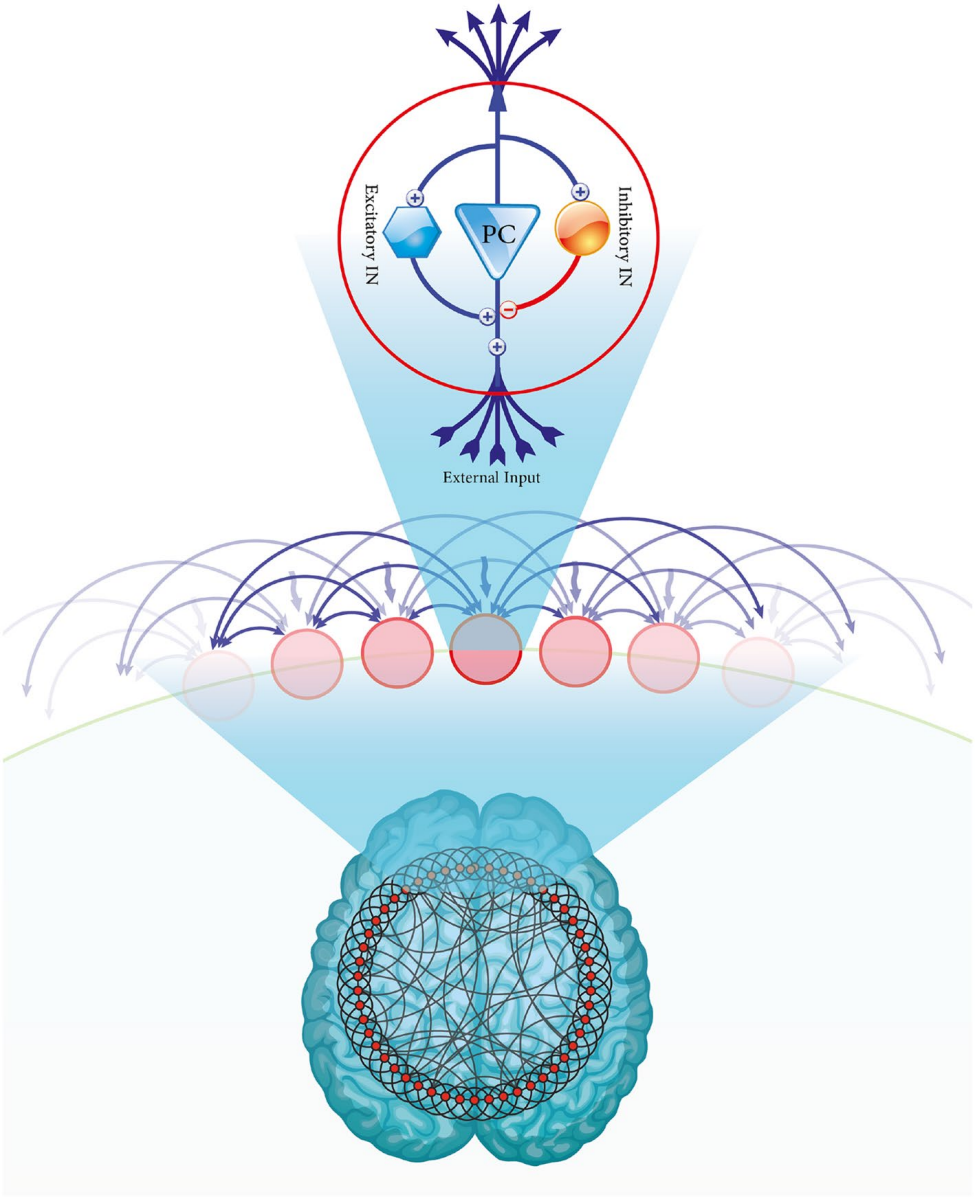
Schematic representation of Jansen–Rit model in a WS network. A main population (pyramidal cell) interacts with two populations: excitatory interneuron (left) and inhibitory interneuron (right).

In this paper, neural masses are defined as columns. The model consists of pyramidal neurons (main population) that receive inputs from three resources: excitatory and inhibitory interneurons feedback in the same column and an external input from other columns. Figure 1 is a schematic representation of this model.

We consider a network with an interactive population, representing a patch of the cortex. Each node indicates a neural mass with Jansen–Rit dynamics. Nodes can interact with each other and generate different behaviors. First, we represent this dynamic per node k, k =1,..., N. The equations are written by three second-order differential equations and can rewrite with six first-order differential equations as follow^39^:

$$\begin{aligned} \begin{array}{ll} {\dot{y}}_{0} (t) = y_{3} (t)\\ {\dot{y}}_{3} (t) = A a S(y_{1} (t) - y_{2} (t)) -2a y_{3} (t) -a^{2}y_{0} (t)\\ {\dot{y}}_{1} (t) = y_{4} (t)\\ {\dot{y}}_{4} (t) = A a \left\{ p(t)+J_{2} S[J_{1} y_{0} (t)] \right\} -2ay_{4}(t) -a^{2} y_{1}(t)\\ {\dot{y}}_{2} (t) = y_{5} (t)\\ {\dot{y}}_{5} (t) = B b J_{4} S(J_{3}y_{0}) -2b y_{5} (t) -b^{2}y_{2} (t) \end{array} \end{aligned}$$

where (*y*0, *y*3) , (*y*1, *y*4) and (*y*2, *y*5) are activities of pyramidal, excitatory and inhibitory ensembles respectively.

S is a sigmoid function, transforms the average membrane potential of neurons to the mean firing rate of action potentials, and is identified as follows:

$$\begin{aligned} S(v) = \frac{v_{max}}{1 + e^{r(v_{0}-v)}} \end{aligned}$$

p($s^{-1}$) means an external input, which can be considered as noise or input from other columns. It used by Jansen and Rit was uniformly distributed noise ranging from 120 to 320 pulses per second^38^.

Other parameters are quantified in Table 1^75–77^. The output of this model is the postsynaptic membrane potential of pyramidal neurons $(y1-y2)$. In terms of the incoming firing rate of pyramidal cells, the variable $y1-y2$ is closely related to the EEG signal. The apical dendrites of pyramidal neurons deliver their postsynaptic potentials to the cortex’s superficial layer, which accounts for the largest portion of the EEG^76^. Also $J_{i} = J \alpha _{i}$, for i =1, ..., 4.

**Table 1:** Parameters in the Jansen–Rit model are obtained experimentally^38^.

Parameters	Interpretation	Value
*A*	Amplitude of excitatory PSP	3.25 mV
*B*	Amplitude of inhibitory PSP	22 mV
1/*a*	Time constant of excitatory PSP	$a = 100~s^{-1}$
1/*b*	Time constant of inhibitory PSP	$b = 50~s^{-1}$
*J*1, *J*2	Average numbers of synapses between excitatory neural populations	$1 * J$, $0.8 * J$
*J*3, *J*4	Average numbers of synapses between inhibitory neural populations	$0.25 * J$
*J*	Average numbers of synapses between neural populations	135
$v_{max}$	Maximum firing rate	5 Hz
*v*0	Potential at half of maximum firing rate	6 mV
*r*	Slope of sigmoid function at *v*0	$0.56~mV^{-1}$

Now, the Jansen–Rit’s dynamical equations in a network with N nodes, i=1,..., N, are as follows^35^:

$$\begin{aligned} \begin{array}{ll} {\dot{y}}_{0_{i}} (t) = y_{3_{i}} (t)\\ {\dot{y}}_{3_{i}} (t) = A a S(y_{1_{i}} (t) - y_{2_{i}} (t)) -2a y_{3_{i}} (t) -a^{2}y_{0_{i}} (t)\\ {\dot{y}}_{1_{i}} (t) = y_{4_{i}} (t)\\ {\dot{y}}_{4_{i}} (t) = A a \lbrace p_{i}(t)+ \alpha \sum _{j=1}^{N} M_{ij} S(y_{1_{j}} (t) - y_{2_{j}} (t)) + J_{2} S[J_{1} y_{0_{i}} (t)]\rbrace -2ay_{4_{i}}(t) -a^{2} y_{1_{i}}(t)\\ {\dot{y}}_{2_{i}} (t) = y_{5_{i}} (t)\\ {\dot{y}}_{5_{i}} (t) = B b J_{4} S(J_{3}y_{_{i}0}) -2b y_{5_{i}} (t) -b^{2}y_{2_{i}} (t) \end{array} \end{aligned}$$

where $M_{ij}$ shows the adjacency matrix of the network and $\alpha$ represents coupling coefficients between nodes.

### Simulation

A Watts–Strogatz network (with a rewiring probability of 0.2) is considered in this simulation with 80 oscillators as nodes. Each node is connected to 20 neighbors, ten on each side. Nodes are represented by using Jansen–Rit’s neural mass model. We use the stochastic Runge–Kutta method to simulate the system^78^. The time step is set to $10^{-4}$ s. The coupling coefficient between neurons in each mass is considered 135 (to show alpha rhythm in every single node). Each node receives external inputs from other brain regions. First, we suppose that the coupling coefficient between nodes is equal to zero, and then we start to increase it. The external input used by Jansen and Rit is a uniformly distributed noise ranging from 120 to 320 pulses per second^38^. We divide this range into four segments. We analyze the network dynamics based on the permitted input intervals for four different inputs: weak, intermediate, strong, and ultra-strong. The input is Gaussian random noise with a mean of 145, 195, 245, and 295 in four intensity levels respectively, and sigma = 3.25 for all of them. The length of this simulation is 200 s and runs 20 times on each coupling coefficient. Elimination of the first 5 s of simulation ensure us that the system has achieved its equilibrium.

## Results and discussion

### Synchronization phase transition

**Figure 2:**
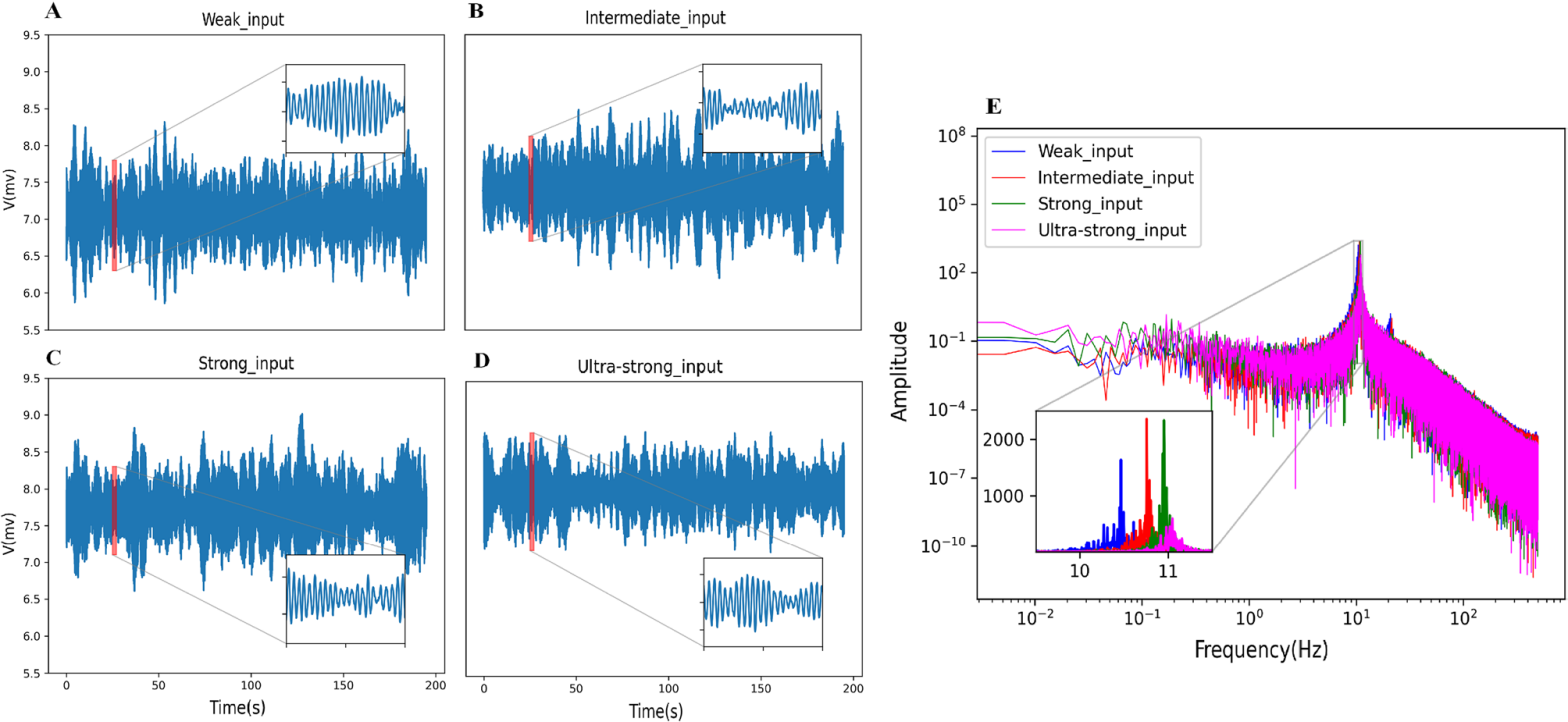
The mean of activity (**A**–**D**) and their power spectrum (**E**) for a single unit in different level inputs ((**A**): weak, (**B**): intermediate, (**C**): strong, (**D**): ultra-strong). Increased input range can increase level activity and dominant frequency (the inset of (**E**)). Alpha rhythm is dominant in all of them (**E**).

We start with a single node that describes the Jansen–Rit model. It is investigated that how the system responds to continuous changes in external input set as deterministic parameters. Note that the external input has been varied in four ranges and regarded as stochastic in this paper. The mean of the output model and its power spectrum for four input levels is shown in Fig. 2. Increased input range can increase the level of activity. In each intensity of input in this figure (A–D), this model produces an oscillatory behavior. Increasing the input increases the dominant frequency, but it is still in the alpha band (E).

Then, we consider a network with an interactive population, representing a patch of the cortex. Each node shows a neural mass with Jansen–Rit dynamics. A number of factors affect network dynamics. One of the most important factors is coupling strength. Nodes can interact with each other and generate different dynamics behaviors. Changing the coupling coefficient between nodes and analysis of network dynamics has received interest recently^51,54,55^.

**Figure 3:**
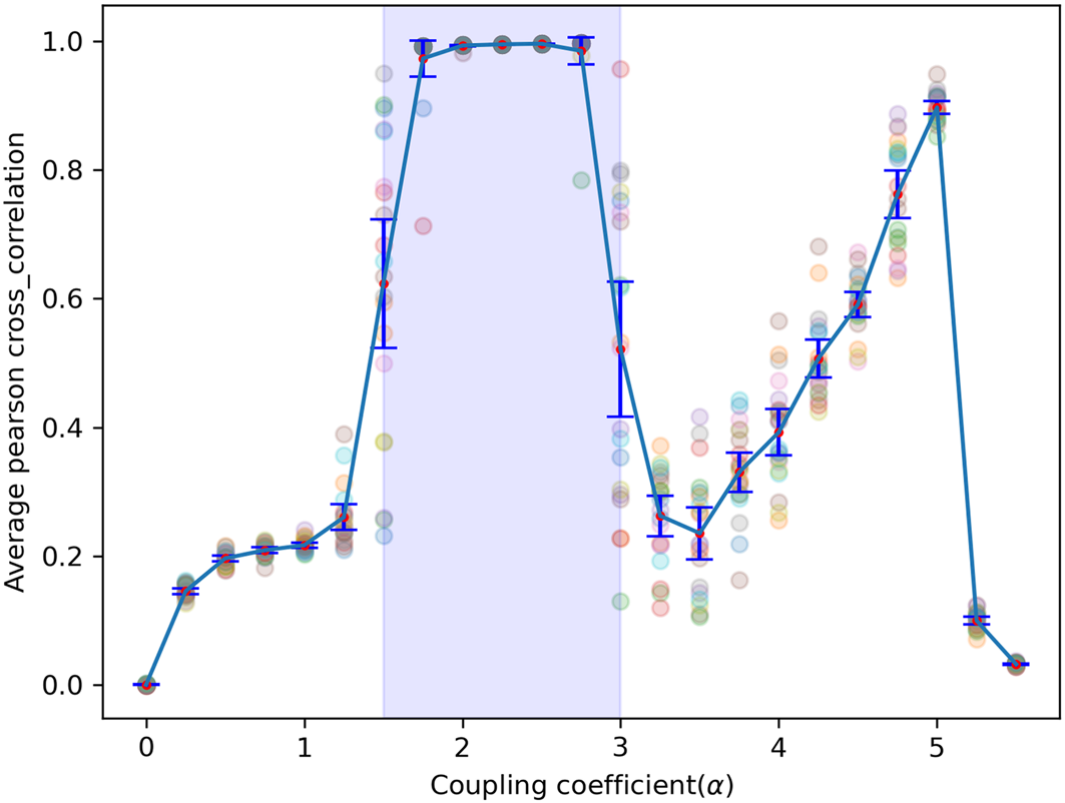
Mean of the Pearson cross-correlation matrix according to the coupling coefficient in a weak input area. In $\alpha$ = 1.5 and 3, the dispersion of the mean of the Pcc is high, and the area between them is related to the high synchrony regime. The simulation runs 20 times on each coupling coefficient.

In the beginning, we assume that each node receives the external input from a weak range. Cross-correlation is a popular method to investigate and compare time series. By considering this time series of each node in the model and calculating the Pearson cross-correlation (Pcc) between them, a square matrix is created. The ij’th element of the matrix shows the Pcc of node i and j dynamics. The mean of the Pcc matrix for weak input is shown in Fig. 3. Vertical red bars represent dispersions for 20 runs (standard deviations).

**Figure 4:**
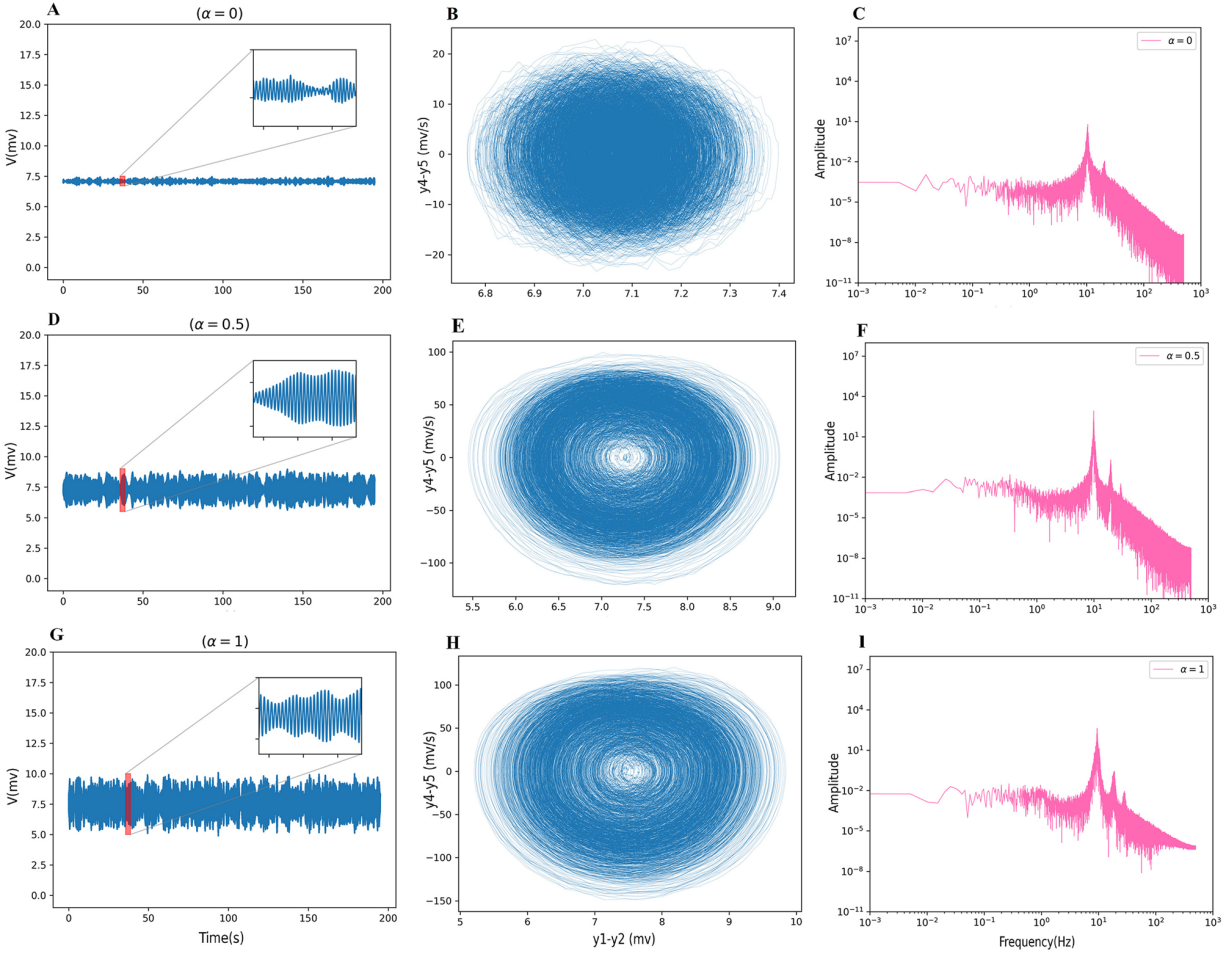
Time series of the mean of the network activity for $\alpha$ = 0 (**A**), 0.5 (**D**), and 1 (**G**) and their phase space (**B**, **E**, **H**). The x-axis and y-axis show the output signal and its derivative, respectively. By increasing the coupling coefficient, the mean signal amplitude gets larger. The inset of (**A**), (**D**) and (**G**) shows a specific segment of signal for 3 s. The power spectrum for these coupling coefficients are shown in the third column. The dominant frequency is in the alpha band.

A zero cross-correlation results from no coupling. As shown in Fig. 3 increasing coupling up to 1.5 causes more correlation. In addition, the mean signal amplitude gets larger (first column in Fig. 4). Their phase spaces are shown in the second column of the Fig. 4. The x-axis and y-axis show the output signal ($y_{1}(t) - y_{2}(t)$) and its derivative ($y_{4}(t) - y_{5}(t)$), respectively. In this range of coupling strength, the dominant frequency is still in the alpha band (third column in Fig. 4).

**Figure 5:**
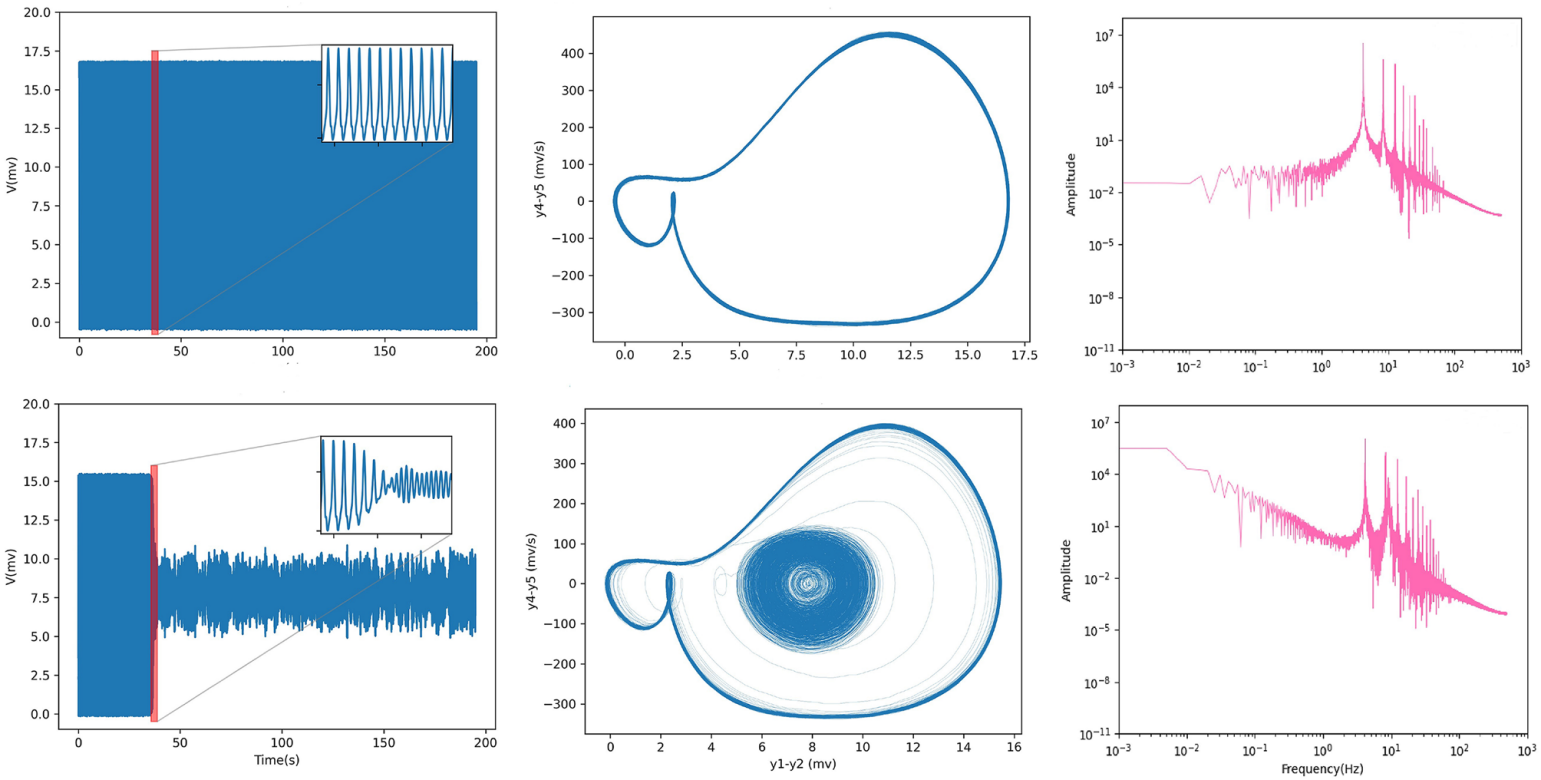
Two types of behaviors between $\alpha$ = 1.5 and $\alpha$ = 3 (first column). Accordingly, one of these states is guaranteed to appear in every repetition of $1.5< \alpha <3$ (regardless of alpha). Two independent runs of $\alpha =1.5$ are presented here. High amplitude oscillations appear in this regime that either change to low amplitude oscillations or not. Their phase spaces are composed of 2 limit cycles or just one limit cycle (second column). The inset of the time series in the first column shows a specific interval of signal for 3 s. The power spectrum for the values of $\alpha = 1.5$ shows a significant change in their appearance (the third column). Theta is the dominant frequency.

At a coupling coefficient equal to 1.5, the network’s behavior abruptly has been changed, and as it can be seen in Fig. 5 (first column), the large amplitude oscillations appear. In each run, switching from high to low amplitude oscillations occurs at different times. In addition, its phase space is shown in Fig. 5 (second column). It is obvious that in this coupling coefficient, the phase space is composed of 2 limit cycles. The first cycle in respect of high amplitude oscillations shows rhythmic spiking activity, and the second corresponding to a shorter amplitude signal. High amplitude signals are rhythmic spiking activities represented in theta band frequency. On the other hand, the dominant frequency of the short amplitude regime is alpha. Indeed, this behavior is a mixed theta-alpha activity. The power spectrum for the value of $\alpha =1.5$ shows a significant change in their appearance (the third column in Fig. 5), which confirms a disorder in network function^79–81^.

Results indicated that in the [1.5, 3] regime of coupling strength, the output signal for all repetitions shows only two types of behavior: high amplitude oscillations that either change to low amplitude oscillations or not (first column in Fig. 5). We call this area a high synchrony regime, and the coupling coefficient equal to 1.5 (3) is the starting (ending) point of unusual behavior.

**Figure 6:**
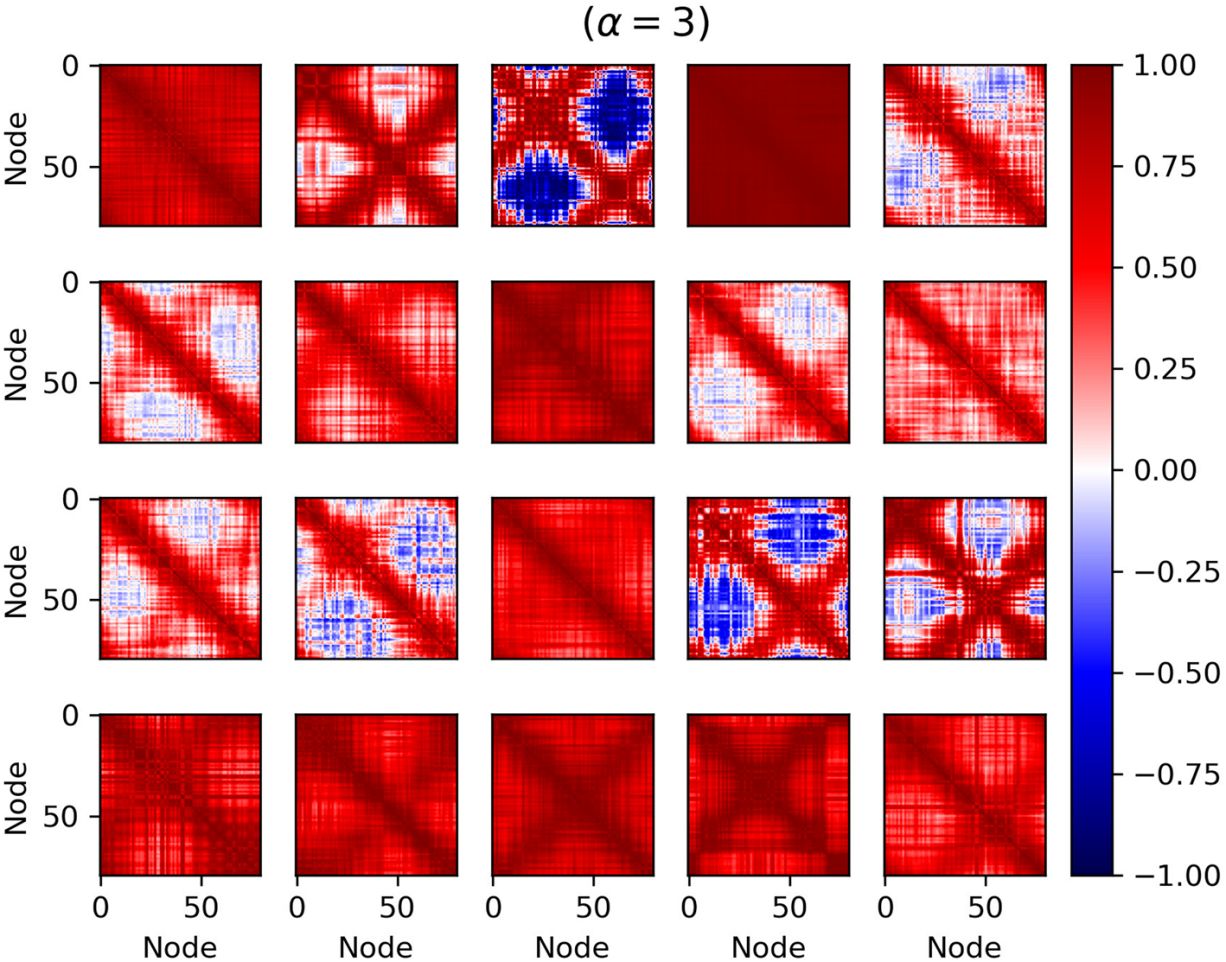
Correlation matrices in $\alpha = 3$ over 20 repetitions during weak input. Local and global synchronizations are seen. During local synchronization, some ensembles of neurons have the same behavior (red color), while others act completely differently (blue color). Full red Pearson cross-correlation matrices mean that the network shows a global synchrony state.

Based on Fig. 3, a minor increase in coupling coefficient of more than 1.5 causes a dramatic rise in the mean of the Pcc, and, consequently, the network is brought into the maximum synchronization. Besides, it can be seen that there is a big dispersion of the mean of the Pcc matrix at bifurcation points (1.5 and 3). In fact, at these points, the network dynamics changes dramatically. A coupling coefficient of 1.5 and 3 yields a large variety in the mean of the Pcc, accompanied by a large variance. Cross-correlation matrices in the value of 3 are shown in Fig. 6. These matrices have different patterns. The red (blue) color shows full synchronization (anti-synchronization). Also, local and global synchronization can be seen in this figure. This issue is specified with red and blue masses, respectively. During local synchronization, some ensembles of neurons have the same behavior, while others act completely differently, i.e., synchronized clusters. To put it simply, full red Pearson cross-correlation matrices mean that the network shows a global synchrony state, and the simultaneous presence of blue and red masses in a matrix is a symbol of in-phase and anti-phase local synchronization. This different pattern could be reminiscent of different synchronization patterns of brain networks during different tasks^54^.

**Figure 7:**
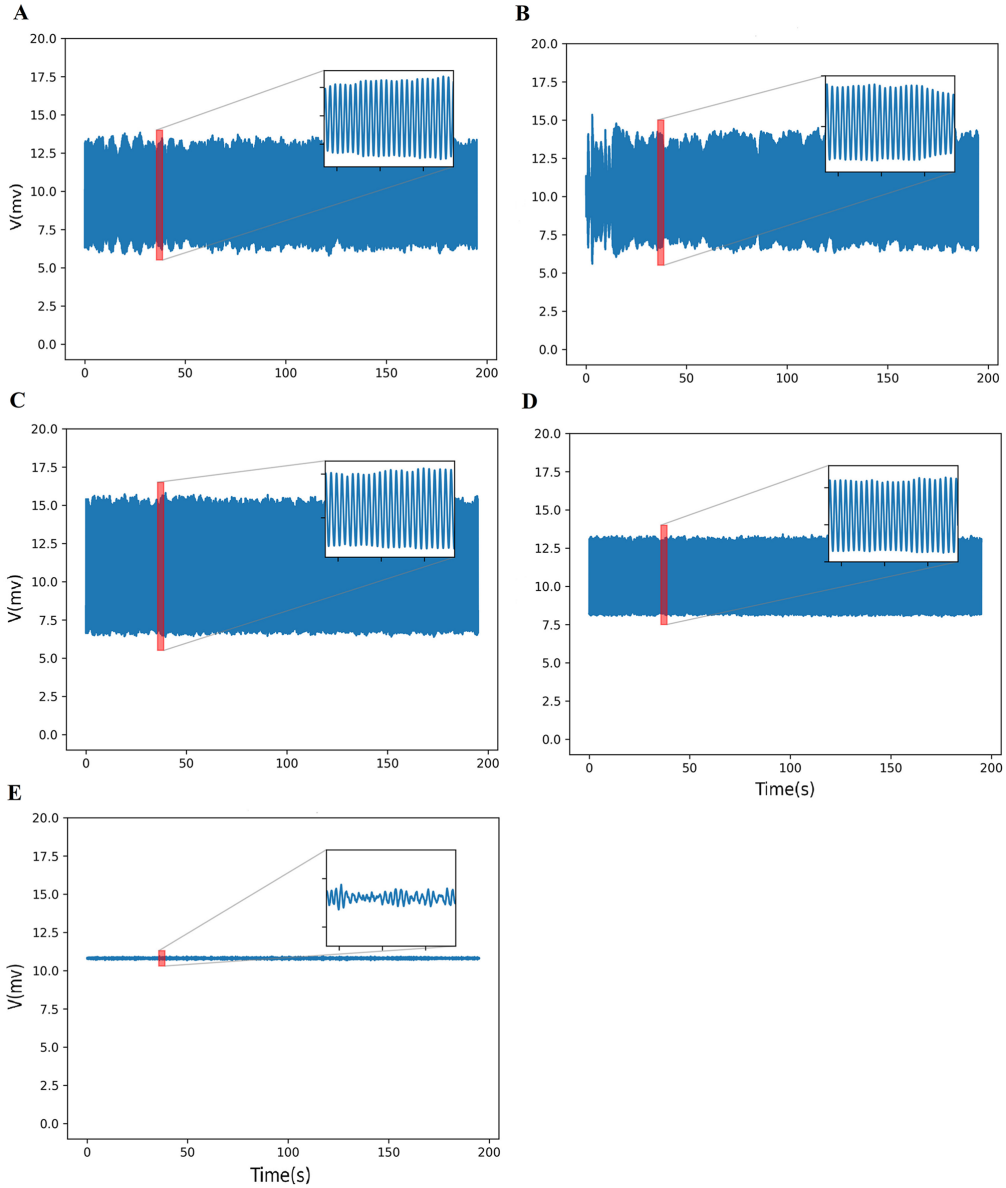
Time series of the mean of the network activity for $\alpha$ = 3.5 (**A**), 4 (**B**), 4.5 (**C**), 5 (**D**), and 5.5 (**E**). The oscillatory activity is seen in (**A**–**D**). The inset of signals shows a specific segment of signal for 3 s. In $\alpha$ = 5.5, each node leaves the limit cycle and take in resting state (fixed-point).

Again, we tracked the increase in coupling strength. A decrease in the mean of the Pcc has been observed up to 3.5. Figure 7 displays that the mean signal output goes back to the oscillatory state.

**Figure 8:**
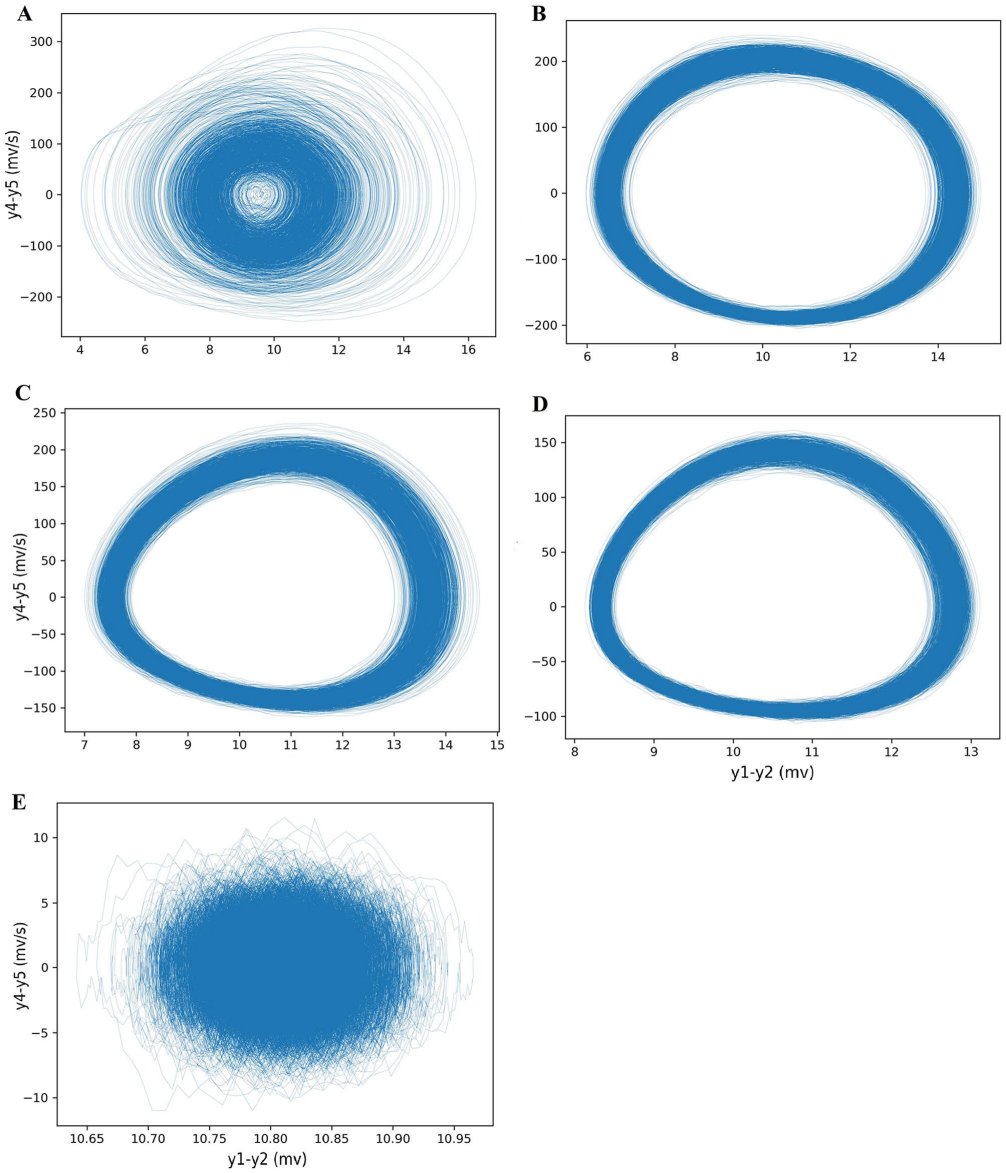
The phase spaces for the time series in Fig. 7 ($\alpha$ = 3.5 (**A**), 4 (**B**), 4.5 (**C**), 5 (**D**), and 5.5 (**E**)). In the [3.5–5], the increase of synchronization is seen. The range of the axis drastically decreases when $\alpha = 5.5$, which indicates the fixed-point state.

As $\alpha$ increases from 3.5 to 5, the Pcc grows to its maximum value. The synchronization behavior switches to a fixed-point state if $\alpha$ is increased further, i.e., each node leaves the limit cycle and collapses into a resting state (fixed-point). Mean signals and their phase space for one repeat are shown in Figs. 7 and 8, respectively.

**Figure 9:**
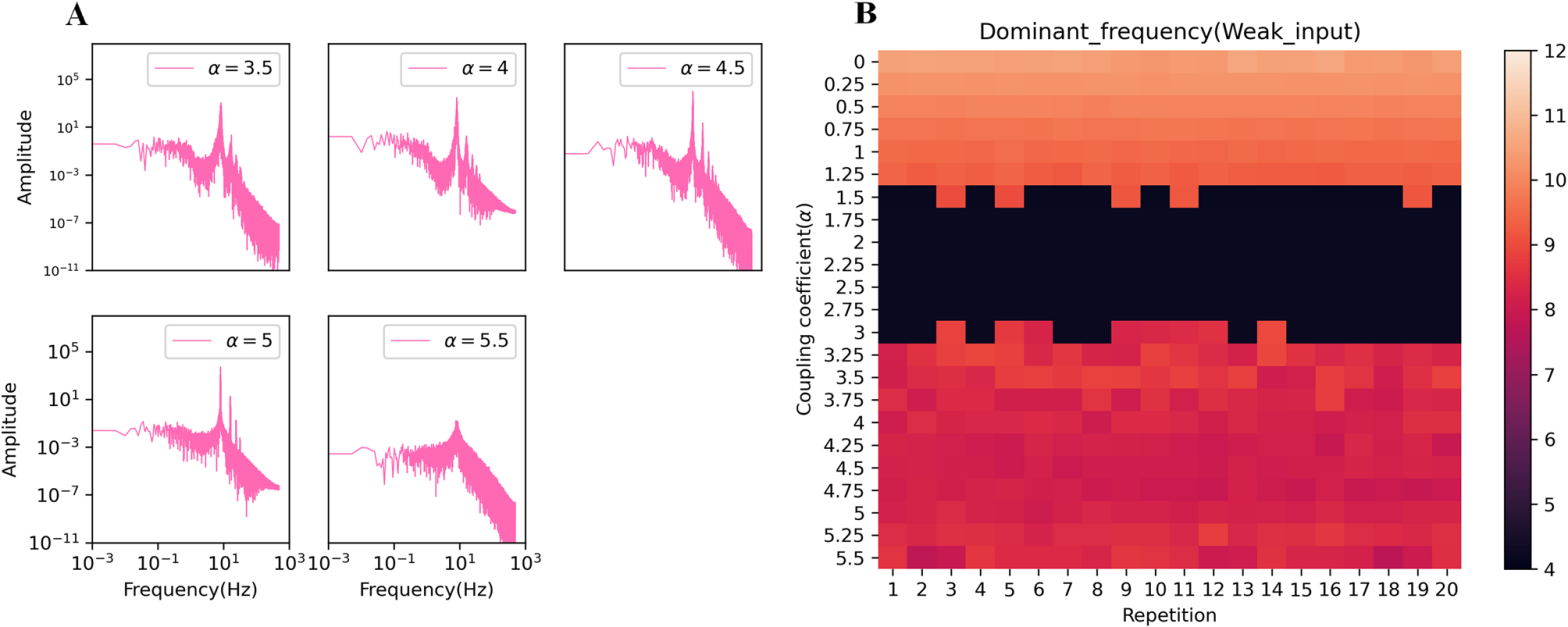
(**A**) Power spectrum for different coupling coefficients in weak noise. The dominant frequency is in the alpha band. At $\alpha$ = 5.5, which indicates the fixed-point state, the power spectrum peak is drastically decreased. (**B**) The value of dominant frequency in different coupling coefficient for 20 runs. A jump phenomenon is seen in frequency in the [1.5–3] regime.

Moreover, their power spectrum for $\alpha =3.5$, 4, 4.5, and 5 in Fig. 9A shows a switch from disorder to the normal state, and the dominant frequency of the network goes into an alpha rhythm. Indeed, in these coefficients, the mean signal output goes back to the oscillatory state. In addition, the theta band frequency changes to the alpha band frequency. The alpha rhythm does not necessarily indicate the healthy state (the average potential value is increased). We just concluded that the system leaves the disorder state. It may enter any pathologic condition. A close look at Fig. 9B shows the dominant frequency based on the different coupling coefficients. The color of each cell represents the dominant frequency of an independent in-silico experiment (run). The vertical axis corresponds to the coupling strength value of each run in its raw. There is a jump phenomenon in frequency in the [1.5–3] regime that is a crucial feature to diagnose the brain disorder^79,82,83^. It is clear that the coupling coefficient and dominant frequency have an inverse relationship. Also, presence of two states in frequency at $\alpha =1.5$ and 3 is an exciting result.

So far, the amount of external input to each node has been considered from a weak level. Next, we look at the input values in the other three ranges (medium, strong, and ultra-strong) and go over the results.

**Figure 10:**
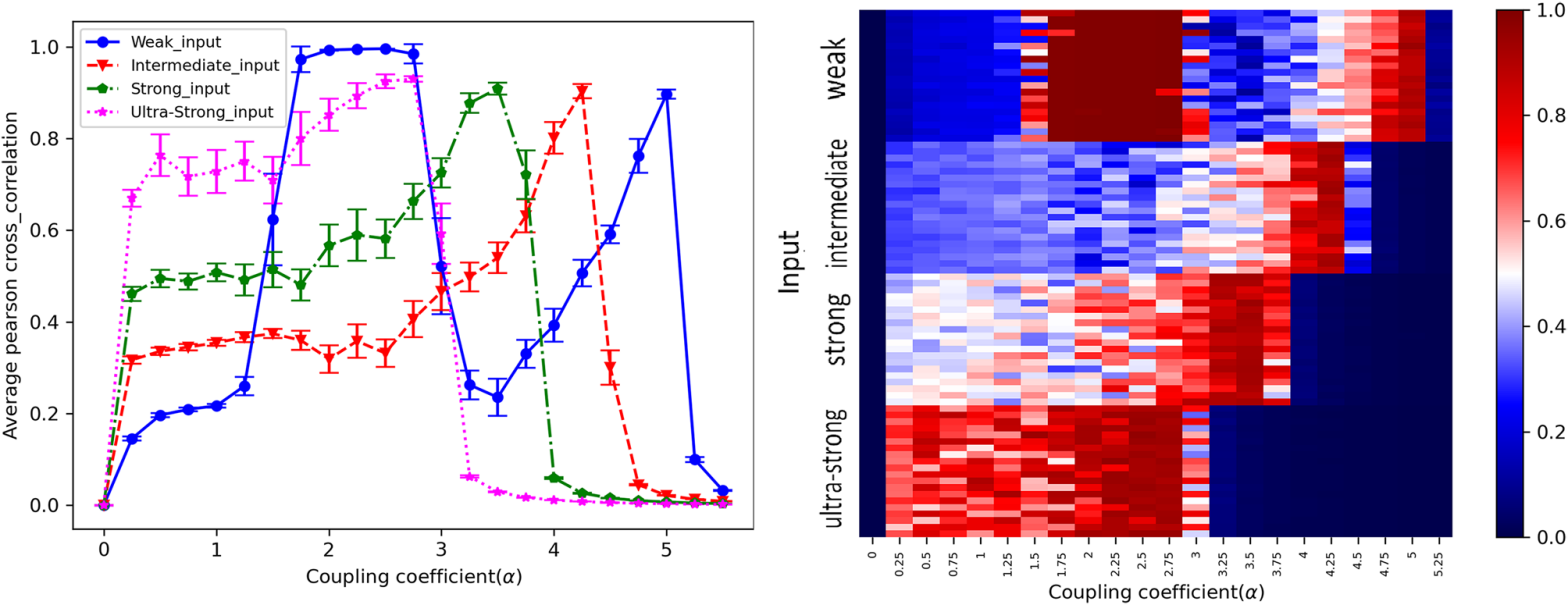
(**A**) A plot of the mean of the Pcc matrix versus the coupling coefficient for four levels of input. The Exception of behavior in the weak input level, lower intensity input needs a stronger coupling coefficient to reach its Pcc maximum. (**B**) The heat-map of the mean of the Pcc matrix for 20 independent runs in each of them, where the x-axis is coupling coefficients, and the y-axis is input signals. Indeed, using Seaborn, a Python data visualization library based on Matplotlib, we visualized the data.

An illustration of the mean of the Pcc matrix plot for the four inputs against coupling strength is shown in Fig. 10A. Moreover, for better understanding, Fig. 10B is a heat-map of the mean of the Pcc matrix for 20 independent runs in each of them, where the x-axis is coupling coefficients, and the y-axis is input signals.

A low correlation between units is observed for all input intensities for zero couplings. In this case, oscillators can not have a significant effect on each other. It is obvious that lower intensity inputs require a higher coupling coefficient to reach their maximum Pcc. Every unit receives some inputs from its neighbors plus the stochastic amount chosen from an interval. Strengthening coupling between nodes leads to receiving more from neighbors, and as a result, less stochastic value is needed. Indeed, the input is a fundamental element, and its variations can affect the amount of correlation and the rate of the upward trend to reach the highest degree of this. Interestingly, though plots in each input intensity have been shifted to the left side, the dispersions of the mean of the Pcc matrix at the $\alpha =1.5$, 3 are still high.

**Figure 11:**
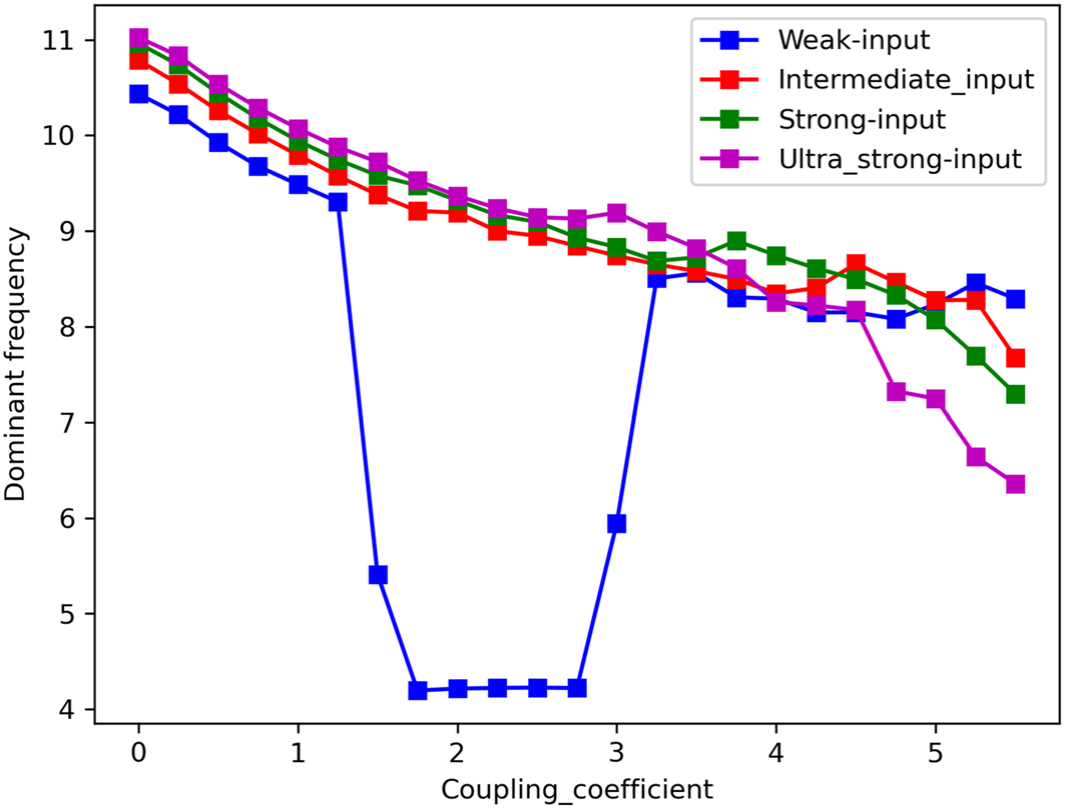
Dominant frequency as a function of the coupling coefficient. An increase coupling coefficient decreases the dominant frequency. One remarkable event is viewed: a jump frequency in the weak level input that is related to the high-frequency regime.

A remarkable event is seen in Fig. 11: a jump frequency in the weak level input that is related to the high-frequency regime and has been confirmed in^79^. Additionally, in each input level (except the weak level), increasing coupling coefficients leads to a decrease in the dominant frequency, while remaining within the alpha band.

### Measure of criticality

It is claimed that the healthy brain acts in a critical regime^64–67^. It is an interesting and challenging question that how critical dynamics can be detected in neural models. There are some measures of criticality that can be tested.

**Figure 12:**
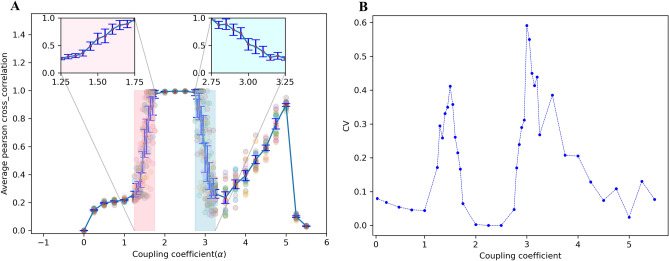
(**A**) The mean of the Pcc matrix as a function of the coupling coefficient. The colored region between 1.25 and 1.75 and between 2.75 and 3.25 are zoomed in the red and blue inset, respectively. (**B**) The coefficient of variation (CV) against the coupling coefficient. These points of maximum of this curve correspond to the value of the transition points in Fig. 12A.

In order for any marker of criticality (observed in the brain) to exist, first a critical point or a critical region (Griffiths phase) needs to be determined. We have investigated several points between 1.25 and 1.75 (1.25, 1.3, 1.35, 1.4, 1.45, 1.5, 1.55, 1.6, 1.65, 1.7, 1.75), and several points between 2.75 and 3.25 (2.75, 2.8, 2.85, 2.9, 2.95, 3, 3.05, 3.1, 3.15, 3.2, 3.25). Figure 12A shows the mean of the Pcc matrix as a function of the coupling coefficient. The colored region between 1.25 and 1.75 and between 2.75 and 3.25 are zoomed in the red and blue inset, respectively. Two insets demonstrate that if a phase transition emerges, it is not the first (discontinuous) phase transition. To investigate the second phase transition, we compute the coefficient of variation (CV) of Fig. 12A against the control parameter (coupling coefficient). Two peaks in the coefficient of variation during the continuous phase transition (in $\alpha =1.5$, 3) are the first marker of criticality (Fig. 12B)^57^. These points of maximum of this curve correspond to the value of the transition points in Fig. 12A.

**Figure 13:**
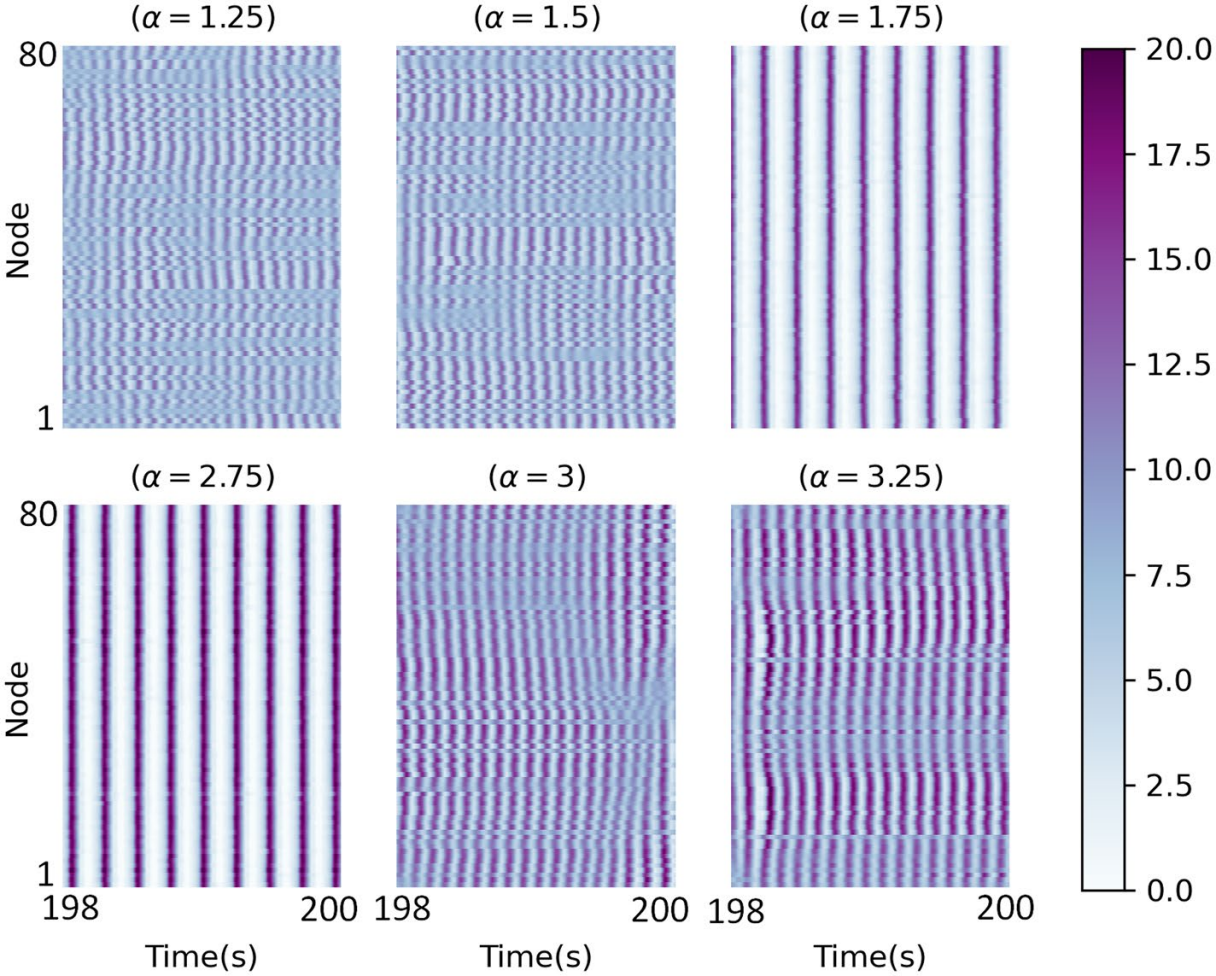
The spatiotemporal matrices of the mean signal for different values of coupling coefficient. In $\alpha 1.25$ (3.25), the fluctuations are random, and the value of synchronization is low. Conversely, in $\alpha = 1.75$ (2.75), the activity of the network is extremely ordered. In $\alpha =1.5$ (3), there is a phase transition from disordered (ordered) to ordered (disordered) states, and the system in these coupling strengths is being on the edge of phase synchronization.

The expression of the criticality hypothesis is that the brain might work at the edge of a phase transition^57^. Our research considers synchronization phase transition, so we studied $\alpha =1.5$, 3 candidate points that can be validated as critical. On the edge of synchronization, a different range of patterns can be generated, as shown in^84^. A heatmap of the spatial-temporal matrix during the last 2 s in $\alpha =1.25$, 1.5, 1.75, 2.75, 3 and 3.25 is shown in Fig. 13. In $\alpha =1.25$, the fluctuations are random, and the value of synchronization is low. Conversely, in $\alpha = 1.75$, the activity of the network is extremely ordered. This pattern is related to full synchronization that does not show a healthy state. Within this range, in $\alpha =1.5$, the network activity is between high and low synchrony. Similarly, in $\alpha =2.75$ and 3.25, activities are highly ordered and disordered, respectively, and in $\alpha =1.5$ and 3, the network shows a pattern between ordered and disordered states. So, it is possible to say that these points (1.5 and 3) have a marker of criticality.

At critical points, the perturbations grow in magnitude, and different behavior occurs. Since the system stability is weak, it is expected to see events in each scale (micro or macro) that it is one of the most remarkable properties in criticality. Broadly speaking, the border between scales is not clear. Dysfunction of the cortex is associated with either abnormally low or high synchrony. Moderate synchrony is characteristic of a healthy cortex. In some cases, extreme variability in synchrony is unavoidable if the cortex must operate with moderate mean synchrony. At critical points, synchrony has a medium mean and high variability^85,86^. Due to this, both $\alpha =1.5$ and 3 have a criticality marker as they satisfy this criterion (based on Fig. 3)^57,68,87,88^.

The presence of LRTCs in the amplitude of neural oscillations supports the critical hypothesis. Indeed, long-range temporal correlations are a vital feature of criticality^89,90^.

The temporal correlation structures of the signal are investigated by the mean auto-correlation or DFA method. A signal can show LRTCs if its auto-correlation decays as a power law (with an exponent between $-1$ and 0). Generally, auto-correlation functions are very noisy in their tail, and so, the exponent estimation is very complicated. DFA is a proper technique that overcomes these problems^90^. This technique in neural mass models is used recently in^51^.

**Figure 14:**
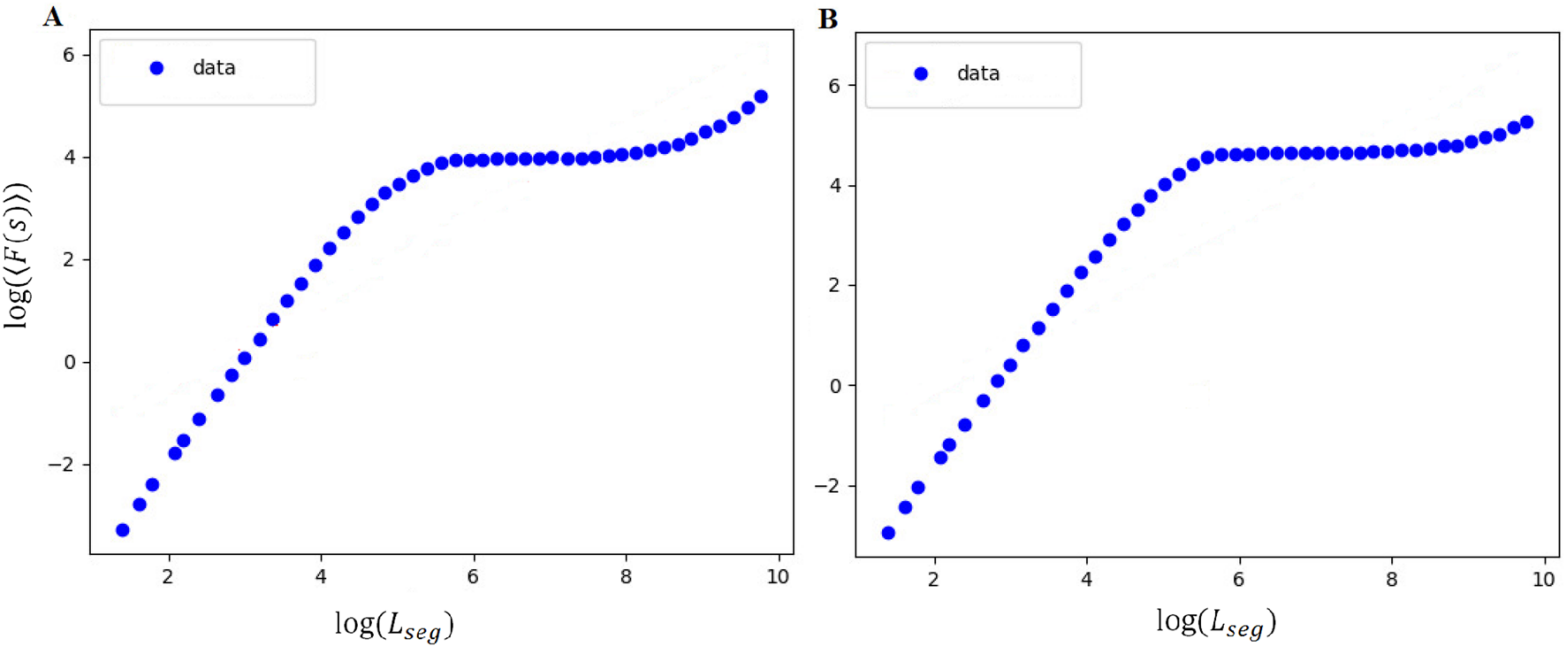
The fluctuation plot for $\alpha =1.5$ (**A**) and $\alpha =3$ (**B**) in the weak input level in the log–log plot. The values on the x-axis are in seconds on logarithmic scales based on segment sizes, and the y-axis shows the standard deviation mean of all sized segments. This trend of data is piecewise linear, and the linear fit is not suitable for them. So, LRTCs do not exist at these points.

According to the previous section, it seems that one phase transition occurs in the weak input level around $\alpha =1.5, 3$. We checked the presence of LRTCs at these points in an arbitrary repetition. In Fig. 14 the DFA is applied in an absolute of signal Hilbert transformation with no overlapping. The values on the x-axis are in seconds on logarithmic scales based on segment sizes ($L_{seg} = 2N_{s}$ and the definition of $N_{s}$ can be found in the “Methods” section). The y-axis shows the standard deviation mean of all sized segments (*F*(*s*) is relevant to the given definition of the “Methods” section). The linear fit is not suitable for data (on the report of^91^ spline fit is the best fit model). So, LRTCs do not exist at these points. Similarly, LRTCs have not been exhibited in other coupling coefficients, and consequently, the network with defined parameters does not show this feature.

### Link to seizure

Seizure is characterized by complex dynamics, and it is a result of a high level of neuronal synchrony. The transition between high and low amplitude signals is one of the most indicators of brain disorders such as epilepsy^79,92,93^. A generalized spike-wave discharge (GSWD) occurs at $\alpha = 1.5$ with a frequency band of approximately 4 Hz. Commonly, GSWDs have arisen after paroxysmal oscillations in the corticothalamic networks but actually, their process is not clear yet^94,95^. Signals with a high amplitude correspond to rhythmic spikes in theta band frequency. These can indicate seizures, which are confirmed by seizure frequencies^92^. Indeed, the onset of this disorder can be viewed as $\alpha = 1.5$. Regarding coupling strengths between $\alpha =1.5$ and $\alpha =3$, there are two possible behaviors (Fig. 5). Short amplitude signals have presented the alpha rhythm. In mixed seizure-alpha behavior, ictal activity may occur^61^. Importantly, the length of this simulation is 200 s. Indeed, Here, there is seizure activity that lasts up to 200 s or changes to a low amplitude oscillatory state. Consequently, we call this area a seizure regime and the coupling coefficient equal to 1.5 (3) is the starting (ending) point of seizure. Insightful to note that different frequency bands have a remarkable role in seizure^96,97^. In $\alpha =1.5$, we discovered a transition from preictal to ictal state that is recognized by rhythmic spikes in a theta-band frequency and triggers the seizure activity. It is noteworthy the frequency jump is a vital issue to comprehension seizure.

## Conclusion

Neural mass models are widely used to simulate the activity of populations of neurons. In this work, we first explained the one-column Jansen–Rit neural mass model. Next, we constructed a small-world network consisting of identical oscillators that their dynamics corresponded to this model. Our results showed that in a weak input level, the dominant frequency of a single unit would be different from a network of units.

Spiegler et al.^58^ demonstrated that the dominant rhythm could be scaled by the ratio between the inhibitory and excitatory time constants. Our results suggested that even though that the alpha rhythm was prominent in each node, the network comprised of single nodes did not necessarily show this rhythm. This consequence is an emergent property in a complex system. i.e., every isolated node represents an alpha rhythm, which changes when these units interact with each other in a complex system^98^.

Synchronization phenomena have a significant impact on how the brain functions normally and abnormally. Nonlinear dynamics is one of the most fundamental phenomena in phase synchronization. The input level and coupling coefficient are crucial parameters in system synchronization, as we explained in detail. In our research, an unusual event was detected in the weak input range, including a high synchrony activity related to the first peak of the Pcc matrix. $\alpha = 1.5$ and 3 were candidates as bifurcation points, and [1.5-3] was defined as a seizure area. We observed GSWDs with approximately 4 Hz frequency band in $\alpha = 1.5$. It is a vital fact to note that different frequency bands can have significant effects on epilepsy^96^. A transition from a preictal to an ictal state has been observed in $\alpha = 1.5$. A rhythmic spike in theta band frequency marks the beginning of this transition, which triggers seizure activity. Understanding epilepsy involves understanding frequency jump, which is a crucial factor^97^.

Thus far, all of these results have been received from the considering of the weak input. Although the variance of the mean of Pcc was high in $\alpha = 1.5$ and 3 for intermediate, strong, and ultra-strong input levels, coupling strength and input levels were not able to properly produce all behaviors at the weak input level. So no bifurcation and phase transition exists in these cases.

In the final section, we presented some markers of criticality and investigated them at bifurcation points. Criticality is an asserted assumption in a healthy brain. From a theoretical perspective, a system is poised at the critical point or not. However, previous resting-state fMRI studies have shown that the brain spends most of its time wandering around a broad region near a critical point, rather than sitting at it^99^. Indeed, there is a whole extended region around the critical point where cortical networks operate. Due to the complex hierarchical-modular structure of cortical networks, a critical point in the brain can extend to a critical-like area that corresponds to a Griffiths phase in statistical mechanics^100^.

According to our study, some markers of criticality can occur at phase transition points, while others may not. This occurrence is a result of the nature of the specific order parameter selected to observe these markers. In fact, The definition of a proper order parameter is crucial and must be defined properly. Our view is that the critical points exhibit clear characteristics and invariance of scale, instead of some types of markers. As a result, $\alpha = 1.5$, 3 are not critical as they show no evidence of scaling invariance. The definition of new measures or observables where scale-invariance can be appreciated can be a challenging question. One different thing is that empirical measures in the actual brain can exhibit some markers of criticality. Also, it is an open question to be solved where the brain operates (if it works in a critical point/region or not) and the type of phase transition it exhibits.

In summary, the randomness of input, initial conditions, noise and coupling strengths in a network can generate different complex behaviors. We also should mention that these behaviors are not just grounds to trigger a seizure activity because understanding the seizure dynamics can not be achieved easily. There are some research reports about the delay and noise in coupled oscillators^51,101–105^. The delay between neural populations can produce interesting and complex dynamics in networks; however, we assumed that in this work, they were zero. Notice, this model is analyzed in the alpha frequency band, i.e., the coupling between excitatory and inhibitory masses in each node was set to 135 in this model. Further studies can focus on the epilepsy state, and their results may be helpful in the detection and treatment of this disease. It is possible that adjusting the standard deviation of stochastic input would have a substantial effect that was discarded here. It may have some important implications if this is considered.

We are left with the question, how the Jansen–Rit model can be modified to show more markers of critical behavior (especially scale-invariant) and, if so, which parameter adjustments (noise, delay, coupling between inhibitory ensembles, etc.) are necessary.

## Methods

### Graph theory

Modeling the brain as a complex network is a powerful mathematical tool to understand the structural and functional of the brain architecture.

Structural brain networks can be shown as graphs that their nodes (vertices) are related to neural elements (neurons or brain regions) and linked by physical connections (edges). Using adjacency matrix (*M*) is a simple method to display a matrix where $M_{ij}=1$, if there is a connection between node i and node j and $Mij = 0$ otherwise. In this work, neural populations are considered as nodes, and edges are interpopulation connections.

The clustering coefficient computes how connected a node’s neighbors join together to make a cluster. This coefficient for each unit i is identified as follows:

$$\begin{aligned} C(i) = \dfrac{2E_{i}}{k_{i}(k_{i}-1)} \end{aligned}$$

where $E_{i}$ is the number of edges between the neighbors of i and $k_{i}$ shows the i degree. This feature in a network is defined as the average clustering coefficient of units.

The shortest path length is the least number of edges between nodes:

$$\begin{aligned} \sum _{i, j \in V} \dfrac{d(i,j)}{N(N-1)} \end{aligned}$$

where V is the set of nodes and N shows the number of nodes in a netwrk. Also, d(i, j) is the shortest path from node i to node j. The average of this property shows the average node to node distance in a graph. This concept represents how rapidly information can be transformed through the network.

Regular, random, and small-world networks are three important network models which have largely been studied so far. In regular networks, each node has exactly the same number of connections and the clustering coefficient is high in these networks. The connections between each node in random topology follow a normal degree distribution with a low level of clustering. The average path length is short (long) in regular (random) systems.

Evidence suggested that the most real-world networks have small-world properties including two independent structural features, namely, short average path length (long-range connections) and high clustering coefficient^71,106–108^. The Watts-Strogatz (WS) models were generated as the simplest networks that have the small-world properties. There are *N* nodes in a regular ring lattice in the WS network, each connected to its nearest neighbors along with a range of *k*, $\frac{k}{2}$ on either side. With probability p, each edge is rewired to a new node. In the case of p = 0 and p = 1, regular and random networks are produced, respectively.

### Pearson cross-correlation

Neural interactions can be measured by multiple criteria, each with its advantages and disadvantages^109^. The simplest measure of non-directed interactions between random variables is Pcc which measures the linear relationship between each pair of random variables. This coefficient removes the temporal structure, so time series are considered as generalizations of random variables. Normalized Pcc coefficient in one-dimensional between two time-series *x* and *y* is defined as follows:

$$\begin{aligned} r = \frac{\sum _{i=1} ^{N}(x_{i} - \langle x \rangle ) (y_{i} - \langle y \rangle )}{\sqrt{\sum _{i=1} ^{N}(x_{i} - \langle x \rangle )^2} \sqrt{\sum _{i=1} ^{N}(y_{i} - \langle y \rangle )^2}} \end{aligned}$$

where N shows the length of the signal and $<x>$ is the mean of time series *x*. The range value of this coefficient varies between $-1$ and 1. The quantity of $1 (-1)$ represents a perfect linear positive (negative) correlation and 0 means no correlation between two series (uncorrelated state). Note that the matrix constructed by this definition is symmetric and elements are one in the main diagonal.

### Detrended fluctuation analysis (DFA)

DFA method specifies the self-affinity of a signal and demonstrates long-range correlation in time series, which was first introduced by Peng et al.^110^. Illustration of this procedure consists of the following five steps.

- Step 1: We consider time-series x with a length of N and calculate the cumulative sum of $x - <x>$ where $<x>$ is the mean signal of *x*:
  $$\begin{aligned} Y_{t} = \sum _{i=1}^{t} (x_{i} - <x>) \end{aligned}$$
  where $$t \in N$$.
- Step 2: $Y_{t}$ is divided into $N_s = (N/s)$ segments with the same size s. It is possible that (*N*/*s*) not to be an integer, so we do this division from the end of signals. Therefore, we have $2N_{s}$ segments. Then, the least-squares fit is calculated by minimizing the squared errors in each segment.
- Step 3: In each segment, the root mean square (RMS) for *Y* is calculated as follow:
  $$\begin{aligned} F(s) =\root \of {\frac{1}{2N_{s}} \sum _{t=1}^{2N_{s}} (Y_{t} - Y_{local}(t))^{2}} \end{aligned}$$
  which $Y_{local}$ shows the local trend.
- Step 4: We repeat step 3 for different interval sizes ranging between 4 and *N*/10 to find the relation between the scaling exponent and the data fluctuation.
- Step 5: We plot *F*(*s*) consistent with s in a logarithmic scale. In power-law relation, $F(s) \sim s^{a}$ which *a*, fluctuation or scaling exponent, is determined as the slope of a straight line fit. $0.5< a < 1$ represents long-range correlation.

We used Nolds package in Python with no overlapping windows to perform this technique (https://pypi.org/project/nolds/).
